# Assessing the adaptive profiles and thermal comfort status of Hararghe Highland sheep through physiological parameters

**DOI:** 10.1016/j.vas.2026.100738

**Published:** 2026-06-15

**Authors:** Mohammed Yousuf, Kefelegn Kebede, Yesihak Yusuf, Gemeda Duguma, Solomon Abegaz

**Affiliations:** a Dambi Dollo University, Department of Animal and Range Sciences, Dambi Dollo, Ethiopia; b Haramaya University, School of Animal and Range Sciences, Haramaya, Ethiopia; c Wollega University, Department of Animal Sciences, Nekemte, Ethiopia; d International Livestock Research institutes, Addis Ababa, Ethiopia

**Keywords:** Heat stress, Indigenous sheep, Thermoregulation, Temperature-humidity index, Ethiopia, Hararghe highland

## Abstract

This study aimed to characterize the adaptive profiles and thermoregulatory status of Hararghe Highland (HH) sheep across two distinct seasons through evaluating physiological parameters. Fifty-four healthy adult HH sheep were evaluated for body surface temperature (BST), rectal temperature (RT), respiratory rate (RR), heart rate (HR), and pulse rate (PR), with measurements taken twice daily during both seasons across three agroecological zones. Ten heat stress indices, including climate-based (THI, THIn, CCCIg, CCCIe, CCCIp) and physiology-based (ECIg, ECIe, ECIp, IHTI, BHTI), were computed to assess the environmental thermal load and its impact on the physiological heat stress responses of HH sheep using linear mixed models. The results indicated significant effects (p < 0.05) of season, period of day, and agro-ecological zone on all physiological parameters, while sex had no significant effect (p>0.05). Afternoon values were consistently higher than morning values across all parameters (BST: 37.6 vs. 36.6°C; RT: 38.1 vs. 37.5°C; RR: 28.8 vs. 23.9 breaths/min; HR: 83.4 vs. 77.3 beats/min; PR: 80.3 vs. 74.2 pulsations/min), and dry-season values exceeded wet-season values for all parameters. Correlational analysis revealed that, except for IHTI and BHTI, most indices showed strong positive associations with physiological parameters (r = 0.63–0.94), confirming their applicability for evaluating environmental heat load in HH sheep. Climate-based indices (THI, THIn, CCCIp) are recommended for on-farm heat stress monitoring without requiring animal measurement. These findings provide a foundation for evidence-based heat stress management and conservation of locally adapted genetic resources under climate change.

## Highlights

• Hararghe Highland sheep exhibit significant seasonal and diurnal variation in physiological parameters.
• Lowland sheep recorded the highest values, indicating greater thermal stress than sheep in highland area.
• Ten heat stress indices were evaluated; most showed strong correlations with physiological parameters.
• Environmental comfort indices (ECIg, ECIe, ECIp) demonstrated the highest predictive accuracy.
• Climate-based indices (THI, THIn, CCCIp) are recommended for on-farm heat stress monitoring.

## Introduction

Sheep play an essential role in the livelihoods of smallholder farmers, they contribute significantly to food security and the cultural and economic stability of rural communities (Yousuf et al., 2025). Sheep provide households with a source of income from sales of meat, wool, and live animals, diversifying income streams and acting as a form of savings and financial security. This economic role helps buffer families against shocks and supports poverty alleviation (Oluwatayo and Oluwatayo, 2018).

Heat stress is a significant impediment to sheep production in tropical and subtropical areas, where increasing ambient temperatures and humidity impair physiological functions, growth, reproduction, and immune competence (Marai et al., 2007). In response to thermal load, sheep activate various adaptive mechanisms to maintain homeostasis, primarily through physiological, behavioural, and morphological adjustments. These mechanisms are crucial for managing heat stress, which arises when an animal cannot adequately dissipate heat produced by its metabolism or gained from the environment (Haile et al., 2019). In Ethiopia, extensive grazing systems expose the majority of sheep to marked seasonal climatic variation. This traditional management system, which relies on natural pastures and minimal inputs, leaves the animals highly vulnerable to climate change-induced stressors such as severe feed and water shortages, particularly during the dry season (Feleke et al., 2016). To increase agricultural productivity, one must consider the interaction between animals and their environment. For the purpose of maximizing livestock production, it is essential to understand the effects of climatic factors on animal physiology. By altering various elements of their physiology and behaviour, animals react differently to extreme temperature fluctuations (Silva et al., 2010). These include alterations in physiological parameters such as rectal temperature, heart rate, respiratory rate, and body surface temperature (Marai et al., 2007). Various factors, including season, age, sex, time of day, physiological stage, exercise, water consumption, feed intake, and digestion, influence physiological parameters in animals (Ribeiro et al., 2018). These factors can significantly alter parameters such as heart rate, respiratory rate, and rectal temperature, which are critical indicators of an animal’s health and adaptability to environmental conditions. The interaction of these factors is intricate and differs among various species, breeds, and environmental conditions.

Indigenous sheep breeds, such as Hararghe Highland (HH) sheep in Ethiopia, possess inherent adaptive traits developed through centuries of natural selection, enabling them to thrive in harsh environments characterized by limited feed resources, heat stress, and water shortages (Tulu et al., 2023). While Tulu et al. (2022) documented that HH sheep lambs tolerate highly saline water with maintained physiological function, their thermoregulatory responses to seasonal heat stress under field conditions remain uncharacterized. Despite their presumed adaptation to hot environments, the physiological responses, thermal tolerance limits, and suitability of different heat stress indices for evaluating heat load in HH sheep have not been systematically characterized under their natural production conditions. Most previous studies in Ethiopia have focused on productive and reproductive traits of indigenous sheep breeds, with limited emphasis on the comparative evaluation of heat stress indicators and their relationship with physiological responses. Evaluation of adaptation traits in animals' normal production environments reflects the true levels of performance in the base population (FAO, 2012).

Furthermore, previous studies have largely relied on single indices such as THI, without systematically comparing multiple climate-based and physiology-based indices. No single heat stress index fully captures both the environmental thermal load and the animal's physiological response; climate-based indices (e.g., THI, THIn) quantify environmental conditions but cannot reflect the animal's internal adaptive state, whereas physiology-based indices (e.g., ECI variants) incorporate measured animal responses but require restraint and specialized equipment. This absence of comparative evaluation limits the ability to identify the most reliable and practically applicable tools for assessing thermal comfort and adaptive capacity in indigenous sheep breeds. To address this gap, the present study systematically characterizes the thermoregulatory responses of HH sheep under field conditions and performs a comparative evaluation of ten heat stress indices, with the objective of identifying the most reliable tools for resilience assessment. The study findings will provide useful information for breeding heat-tolerant sheep, improving heat stress management practices, and conserving adaptive indigenous genetic resources under current climate change scenarios.

## Materials and methods

### Description of study areas

This study was conducted in three districts, namely Bedeno, Goro‑Muti, and Melka‑Balo, which were drawn from the east Hararghe zone of the Oromia Regional State of Ethiopia. The zone is located between 7°31′17″ N and 9°49′16″ N latitudes and 41°10′16″ E and 42°58′51″ E longitudes. The east Hararghe zone has various climatic conditions, ranging from warm lowlands to cool humid highlands. The lowland climatic zone, locally known as Kolla (Gammojjii), covers approximately 30–40% of the total area. Its elevation ranges from 500 m to 1,500 m above sea level. It receives an average annual rainfall of less than 700 mm. The mean annual temperature ranges from 20°C to 28°C. The cool sub‑humid climatic zone, locally called Woinadega (Badda-dare), covers roughly 35–45% of the area, with elevations ranging from 1,500 m to 2,300 m a.s.l. The mean annual rainfall in this zone exceeds 1,200 mm, and the mean annual temperature ranges from 15°C to 20°C. The cool humid climatic conditions, known as Dega (Badda). This zone covers about 15–20% of the total area, with an elevation between 2,300 m and 3500 m a.s.l. It also receives more than 1,200 mm of annual rainfall, with a mean annual temperature of 10°C to 15°C (MOA, 2001; Yasin and Woldemariam, 2023). Crop production and livestock rearing are major economic bases. The dominant food crops grown are sorghum, maize, wheat, barley, pulses, potato, tomato, and groundnuts, while khat and vegetables are the main cash crops. Major livestock reared include cattle, goats, and sheep (EHZLO, 2021).

### Site selection and sampling animals

Three districts from the east Hararghe zone were purposely chosen from three agro‑ecologies (lowland, midland and highland) based on their sheep production potential. Bedeno district (48% midland) was selected to represent the midland zone, Goro‑Muti district (57% highland) to represent the highland zone, and Melka‑Balo district (41% lowland) to represent the lowland zone. From each district, two kebeles (the smallest administrative unit) were chosen for sampling, with selection based on their comparatively larger sheep population sizes within the district. A total of 54 healthy adult indigenous sheep were selected using stratified random sampling, with 18 animals from each agro-ecological zone. All animals were maintained under extensive farming systems within their native environments to preserve natural adaptive responses and ensure the ecological validity of thermal stress assessments. Health status was evaluated through visual examination, and only animals showing no signs of disease or stress were included in the study. Animal age was estimated using dental chronology, with all subjects classified as healthy adults (2-4 years old) to minimize age-related physiological variation, consistent with protocols used (Ribeiro et al., 2008). Before measuring the physiological parameters, the animals were restrained and calmed. All measurements were conducted by trained personnel and researchers using standardized protocols to ensure consistency and accuracy.

### Data collection and technical procedures

#### Data collection schedule and design

Data were collected during two distinct seasons: the dry season (January) and the wet season (July), representing the extremes of annual climatic variation. This seasonal selection captures the maximum environmental variation and thermal stress conditions, following the temporal framework established by Façanha et al. (2023). Measurements were taken twice daily during the morning (06:00–08:00 h, before grazing) and afternoon (12:00–14:00 h, after resting) periods to capture diurnal variation in environmental and physiological parameters. The resulting dataset comprised four representative observation points per animal (dry season morning, dry season afternoon, wet season morning, and wet season afternoon) which served as the units of analysis in the Linear Mixed Model.

### Climatological data

Climatological parameters, specifically air temperature (AT°C) and relative humidity (RH %), were recorded in the animals’ environments using a portable digital thermo-hygrometer (model HTC-1, accuracy: ±1°C for temperature and ±5% for RH; generic supplier). The device was positioned in a shaded location approximately at animal height level to minimize direct solar influence. Environmental measurements were taken simultaneously with physiological data collection during morning and afternoon sampling periods and subsequently associated with the corresponding physiological measurements of each sheep for statistical analysis. Temperature and humidity values recorded at the beginning and end of each sampling session were averaged and subsequently associated with the corresponding physiological measurements of each sheep for statistical analysis of seasonal and diurnal variations.

### Physiological parameter

Five key physiological parameters were measured for each animal during each sampling session using standardized protocols to ensure consistency and accuracy. Body surface temperature (BST,°C) was read using a digital thermometer at the left side of the thorax as reported by Pantoja et al. (2018). Three readings were taken and averaged to ensure accuracy. Rectal temperature (RT,°C) was measured using a digital thermometer. The digital thermometer was inserted into the rectum of each animal, with the bulb in contact with the mucosa, remaining in the rectum until the thermometer made a beep, which was indicative of the temperature stabilization (Ribeiro et al., 2016). The respiratory rate (RR, breaths/min) was determined by means of visual evaluation, observing the movements of the flank, counting for 15 seconds and multiplying by four, determining the breaths per minute as reported by Souza et al. (2014). The heart rate (HR, beats/min) was measured by indirect auscultation of the heart sounds in the laryngo-tracheal region by placing a flexible stethoscope around the heart, then listening and counting the rate per minute. For the sake of time, the number of beats in 20 seconds was recorded, and the values were multiplied by 3 to get the rates per minute of the animal as reported by Ribeiro et al. (2016). The PR was recorded by observing and counting the pulsations per minute by placing the fingers over the jugular vein of the neck for 60 seconds. Before measuring the physiological parameters, the animals were restrained and calmed to minimize stress-induced variations. All measurements were conducted by the same trained enumerator and researcher throughout the study to ensure consistency and reduce observer bias.

### Heat stress index calculation

Several heat stress indicators have been created to evaluate the animals' capacity to adjust to the environmental circumstances common in tropical climates. These indices take into account the physiological and meteorological factors involved in thermal regulation, such as AT, RH, BST, RT, and RR (Souza et al., 2007). The temperature-humidity index (THI), which takes into account both AT and RH, was used to determine the degree of heat stress that the animal endured (Mishra et al., 2009). Among these indices, the thermal stress that the animal is exposed to at any given time and location is measured by Ibéria's heat tolerance index and Benezra's comfort index, which are of particular relevance to researchers (Moraes Júnior et al., 2010). New heat indices that are advised for tropical locations, such as the general comfort climatic conditions index (CCCIg), effective comfort climatic conditions index (CCCIe), practical comfort climatic conditions index (CCCIp), and the general environmental comfort index (ECIg), effective environmental comfort index (ECIe), practical environmental comfort index (ECIp), have been proposed by Silva et al. (2015). Last but not least, the novel heat indices recommended by Silva et al. (2015) were used in pairs. These heat stress indicators were created in order to determine the thermal comfort status of animals raised in tropical climates. The ten indices were purposefully selected to represent two complementary methodological families: (i) climate-based indices (THI, THIn, CCCIg, CCCIe, CCCIp), which rely solely on meteorological inputs (AT and RH) and are therefore practicable without animal handling; and (ii) physiology-based indices (ECIg, ECIe, ECIp, IHTI, BHTI), which incorporate measured animal responses (BST, RT, RR) and thus provide a more direct reflection of the animal’s thermoregulatory state. The selected indices represent both climate-based (e.g., THI, THIn) and physiology-based (e.g., ECI variants) approaches. This combination allows comparison between indices that estimate environmental heat load and those that directly reflect animal physiological response, thereby enabling evaluation of their relative applicability under smallholder production systems. Evaluating both families simultaneously allows a direct comparison of their predictive performance for HH sheep and provides a basis for recommending the most appropriate index for routine use under Ethiopian field conditions. According to the approaches defined by Haile et al. (2019), Pantoja et al. (2018), Silva et al. (2015), Alam et al. (2011), Mishra (2009), and Mader et al. (2006), ten heat stress indicators were determined using the collected climatological and physiological variables.(Table 1).

**Table 1:** Methods of computing the heat stress indices.

Heat stress indices	Measurements	References
THI	THI = AT°C- {(0.31- 0.31 RH) (AT°C-14.4)}. The values obtained indicate the following: < 22.2 = absence of heat stress; 22.2 to < 23.3 = moderate heat stress; 23.3 to < 25.6 = severe heat stress and ≥ 25.6 = extreme severe heat stress.	Mishra (2009); Alam et al. (2011)
IHTI	IHTI = 100 - [18(RT - 39.1)]. The higher the IHTI value the more heat tolerant the animal were considered.	Mader et al. (2006)
BHTI	BHTI = RT/RR + 39.1/27. The lower the value the more heat tolerant the animal were considered.	Mader et al. (2006)
THIn	THIn = 0.8*AT+RH/100*(AT-14.4) + 46.4. The value 70 or lower values are considered as comfortable, 75 - 78 is stressful, and values higher than 78 are distressful.	Haile et al. (2019); Mader et al. (2006)
CCCIg	CCCIg = - 0.0470*RH + 0.6052*AT- 0.0534*RH + 0.0946*RH + 0.3225*AT (values up to 23.78 = comfort, from 23.79 to 28.26 = danger, from 28.27 to 32.75 = thermal stress, above 32.75 = emergency).	Silva et al. (2015); Pantoja et al. (2018)
ECIg	ECIg = - 0.0656*RT + 0.9173*BST+0.1822*RR (values up to 31.44 = comfort, from 32.45 to 35.68 = danger, from 35.69 to 38.93 = thermal stress, above 38.93 = emergency).	Silva et al. (2015); Pantoja et al. (2018)
CCCIe	CCCIe = -0.0309*RH + 0.6493*AT + 0.3330*BST (values up to 25.66 = comfort, from 25.67 to 30.11 = danger, from 30.12 to 34.56 = thermal stress, above 34.56 = emergency).	Silva et al., 2015; Pantoja et al., 2018
ECIe	ECIe = - 0.0660*RT + 0.9144*BST + 0.1865*RR (values up to 32.46 = comfort, from 32.47 to 35.71 = danger, from 35.72 to 38.97 = thermal stress, above 38.97 = emergency).	Silva et al. (2015); Pantoja et al. (2018)
CCCIp	CCCIp = 0.0571*RH + 1.0480*AT (values up to 34.65 = comfort, from 34.66 to 38.02 = danger, from 38.03 to 41.39 = thermal stress, above 41.39 = emergency)	Silva et al. (2015); Pantoja et al. (2018)
ECIp	ECIp = 0.8854*BST + 0.1695*RR (values up to 33.55 = comfort, from 33.56 to 36.67 = danger, from 36.68 to 39.79 = thermal stress, above 39.79 = emergency).	Silva et al. (2015)

AT = air temperature, RH = relative humidity, BST = body surface temperature, RT = rectal temperature, RR = respiration rate, THI= Temperature-humidity index, IHTI= Iberia Heat tolerance index, BHTI= Benezra heat tolerance index, THIn= Thermal heat index, CCCIg= General comfort climatic conditions index, ECIg= General environmental comfort index, CCCIe= Effective comfort climatic conditions index, ECIe= Effective environmental comfort index, CCCIp= Practical comfort climatic conditions index ECIp = Practical environmental comfort index

### Data analysis

All statistical analyses were performed using SAS software, version 9.4 (SAS Institute Inc, 2016). Climatological variables (AT and RH) and physiological variables (BST, RT, RR, HR, and PR) were analyzed using the Linear Mixed Model (MIXED) procedure to account for repeated measurements taken from the same animals across different seasons and periods. In this model, sex, season, period, and agroecology were included as fixed effects, while Animal ID was included as a random effect to account for the correlation among repeated observations from the same individual. Heat stress indices (THI, IHTI, BHTI, THIn, CCCIg, ECIg, CCCIe, ECIe, CCCIp, and ECIp) were analyzed using descriptive statistics procedures and expressed as means ± standard error. The significance of the fixed effects was tested using the F-test. Post-hoc comparisons for all significant main effects were performed using Tukey’s Honest Significant Difference (HSD) test at a 5% probability level. Relationships between physiological variables and heat stress indices were quantified using Pearson's correlation coefficients.

The following linear mixed model was fitted:

$$\text{Model}:\;Y_{ijklm}=\mu +S_{i}+N_{j}+P_{k}+A_{l}+u_{m}+e_{ijklm}$$

Where: Y_ijklm_ = observed measurement values for climatological variables and physiological parameters

μ = overall population mean
S_i_ = fixed effect of the ith sex (i = male, female)
N_j_ = fixed effect of the jth season (j = dry, wet)
P_k_ = fixed effect of the kth period of day (k = morning, afternoon)
A_l_ = fixed effect of the lth agro-ecological zone (l = highland, midland, lowland)
u_m_ = random effect of the mth animal (to account for repeated measures)
e_ijklm_ = random residual error

## Results

### Climatological variables

The average values of climatological variables measured during the study period are presented in Table 2. The overall mean air temperature (AT) and relative humidity (RH) were 26.1°C and 53.2%, respectively, with coefficients of variation of 27.2% and 32.9%. According the present study, both climatological variables were significantly affected by season, period of day, and agro-ecological zone (*p*<0.05). The mean AT values were significantly higher (*p*<0.05) in the afternoon and during the dry season when compared to morning and wet season, whereas the mean RH values were significantly higher (*p*<0.05) in the morning and during the wet season when compared to afternoon and dry season, respectively. Across agro-ecological zones, AT decreased progressively from lowland to highland, while RH showed the opposite trend.

**Table 2:** The average values of climatological variables by the effect of season, period, and agroecology recorded during sampling.

Factors	Climatological variables	
	Air temperature (°C)	Relative humidity (%)
Overall	26.1±0.48	53.2±1.19
CV %	27.2	32.9
Season		
Dry	27.5^a^ ± 0.36	41.3^a^ ± 0.56
Wet	24.7^b^ ± 0.36	65.2^b^ ± 0.56
Period		
Morning	23.3^a^ ± 0.28	58.1^a^ ± 0.48
Afternoon	28.9^b^ ± 0.28	48.4^b^ ± 0.48
Agroecology		
Lowland	33.3^a^ ± 0.44	38.5^a^ ± 0.69
Midland	25.6^b^ ± 0.44	57.8^b^ ± 0.69
Highland	19.5^c^ ± 0.44	63.4^c^ ± 0.69

^a, b, c^ Means within a column with different superscripts differ significantly at *p*<0.05; CV = coefficient of variation

### Physiological parameter

The average values of the physiological variables of HH sheep as affected by sex, season, period of the day, and agro-ecological zone are presented in Table 3. The overall means for BST, RT, RR, HR, and PR were 37.1°C, 37.8°C, 26.3 breaths/min, 80.3 beats/min, and 77.3 pulsations/min, respectively. Sex had no significant effect (*p*>0.05) on physiological variables (BST, RT, RR, HR, and PR). All physiological parameters (BST, RT, RR, HR, PR) were significantly affected by season (*p*<0.05), with higher values recorded during the dry season than the wet season. The period of the day also had a significant effect (*p*<0.05), with afternoon measurements showing higher values than morning measurements. Agroecology significantly influenced all parameters as well (*p*<0.05).

**Table 3:** The average values of physiological parameters of HH sheep affected by sex, season, period of the day, and agro-ecological zone.

Factors	BST	RT	RR	HR	PR
Overall	37.1±0.07	37.8±0.05	26.3±0.27	80.3±0.29	77.3±0.28
CV (%)	2.58	2.11	15.0	5.30	5.34
**Sex**					
Female	37.0±0.06	37.7±0.04	26.1± 0.19	80.1±0.19	77.2±0.19
Male	37.1±0 .08	37.8±0.06	26.5± 0.26	80.6±0.27	77.3±0.26
**Season**					
Dry	37.3^a^±0.07	38.0^a^±0.05	27.2^a^ ± 0.22	81.5^a^±0.23	78.1^a^±0.22
Wet	36.9^b^±0.07	37.6^b^±0.05	25.4^b^ ± 0.22	79.3^b^±0.23	76.4^b^±0.22
**Period**					
Afternoon	37.6^a^±0.06	38.1^a^±0.05	28.8^a^±0.22	83.4^a^ ± 0.21	80.3^a^ ± 0.20
Morning	36.6^b^±0.06	37.5^b^±0.05	23.9^b^±0.22	77.3^b^ ± 0.21	74.2^b^ ± 0.20
**Agroecology**					
Midland	37.1^c^±0.08	37.5^b^ ± 0.06	26.2^c^±0.27	79.2^b^±0.27	76.4^b^±0.27
Highland	36.5^b^±0.08	37.4^b^ ± 0.06	24.0^b^±0.27	78.9^b^±0.27	75.8^b^±0.27
Lowland	37.7^a^±0.08	38.5^a^ ± 0.06	28.8^a^±0.27	82.9^a^±0.27	79.6^a^±0.27

^a,b,c^ Means within a column and factor bearing different superscript letters differ significantly (*p*<0.05); NS = non-significant at (*p*<0.05; BST = body surface temperature (°C), RT = rectal temperature (°C); RR = respiration rate (breaths per minute); HR = heart rate (beats per minute); PR = pulse rate (pulsations per minute); CV = coefficient of variation

### Heat stress indices

Table 4 shows the average values of the heat stress indicators for HH sheep, which were recorded during different periods of the day across the dry and wet seasons. All heat stress indices were significantly influenced by the period of the day in both seasons (*p*<0.05). Afternoon measurements consistently showed higher mean values for THI, THIn, CCCIg, CCCIe, CCCIp, ECIg, ECIe, and ECIp compared to morning measurements. In contrast, IHTI and BHTI were significantly lower in the afternoon than in the morning. Seasonal variation was also significant (*p*<0.05), with mean THI, THIn, CCCIg, CCCIe, CCCIp, ECIg, ECIe, and ECIp were higher during the dry season in both morning and afternoon periods, whereas IHTI and BHTI were higher during the wet season.

**Table 4:** The average value of heat stress indices of HH sheep across different seasons and periods of the day.

Heat stress indices	Dry season morning	Dry season afternoon	Wet season morning	Wet season afternoon
THI	22.9^a^ ± 0.71	26.8^b^ ± 0.65	20.9^a^ ± 0.68	25.8^b^ ± 0.70
IHTI	125.1^a^ ± 1.81	115.1^b^ ± 1.69	133.1^a^ ± 1.57	121.0^b^ ± 1.89
BHTI	2.98^a^ ± 0.02	2.76^b^ ± 0.02	3.14^a^ ± 0.04	2.81^b^ ± 0.02
THIn	66.3^a^ ± 0.73	70.6^b^ ± 0.73	63.9^a^ ± 0.64	68.6^b^ ± 0.70
CCCIg	23.0^a^ ± 0.84	28.0^b^ ± 0.84	20.2^a^ ± 0.74	25.7^b^ ± 0.81
ECIg	35.8^a^ ± 0.15	37.6^b^ ± 0.15	35.1^a^ ± 0.14	37.0^b^ ± 0.14
CCCIe	28.4^a^ ± 0.61	32.2^b^ ± 0.61	26.2^a^ ± 0.54	30.4^b^ ± 0.59
ECIe	35.8^a^ ± 0.15	37.6^b^ ± 0.15	35.1^a^ ± 0.14	37.0^b^ ± 0.14
CCCIp	26.1^a^ ± 0.95	31.6^b^ ± 0.95	22.9^a^ ± 0.84	29.0^b^ ± 0.91
ECIp	36.8^a^ ± 0.15	38.5^b^ ± 0.15	36.1^a^ ± 0.13	37.9^b^ ± 0.14

^a, b^ Means with different superscripts within the same row in the period of day and season of the year are significant (*p*<0.05); THI =temperature-humidity index; IHTI = Iberia heat tolerance index; BHTI = Benezra heat tolerance index; THIn = thermal heat index; CCCIg = general comfort climatic conditions index; ECIg = general environmental comfort index; CCCIe = effective comfort climatic conditions index; ECIe = effective environmental comfort index; CCCIp = practical comfort climatic conditions index; ECIp = practical environmental comfort index

### Correlation between physiological variables and heat stress indices

Correlation analysis revealed significant associations (p < 0.01) between all measured physiological variables and heat stress indices (Table 5). Most heat stress indices (THI, THIn, CCCIg, CCCIe, CCCIp, ECIg, ECIe, ECIp) showed strong to very strong positive correlations with all physiological parameters (r = 0.63–0.94, *p*<0.01). The environmental comfort indices (ECIg, ECIe, ECIp) had the highest correlations with BST and RR (r = 0.90–0.94). In contrast, IHTI and BHTI demonstrated strong negative correlations (*p*<0.01) with all physiological parameters, suggesting an inverse relationship.

**Table 5:** The correlation between the physiological variables and heat stress indices of HH sheep.

Heat Stress Index	Physiological variables				
	BST	RT	RR	HR	PR
THI	0.69^⁎⁎^	0.68^⁎⁎^	0.73^⁎⁎^	0.66^⁎⁎^	0.66^⁎⁎^
IHTI	-0.74^⁎⁎^	-1.00^⁎⁎^	-0.63^⁎⁎^	-0.60^⁎⁎^	-0.61^⁎⁎^
BHTI	-0.60^⁎⁎^	-0.50^⁎⁎^	-0.97^⁎⁎^	-0.67^⁎⁎^	-0.66^⁎⁎^
THIn	0.66^⁎⁎^	0.64^⁎⁎^	0.71^⁎⁎^	0.63^⁎⁎^	0.63^⁎⁎^
CCCIg	0.70^⁎⁎^	0.70^⁎⁎^	0.74^⁎⁎^	0.68^⁎⁎^	0.67^⁎⁎^
ECIg	0.94^⁎⁎^	0.73^⁎⁎^	0.91^⁎⁎^	0.77^⁎⁎^	0.76^⁎⁎^
CCCIe	0.73^⁎⁎^	0.72^⁎⁎^	0.75^⁎⁎^	0.69^⁎⁎^	0.69^⁎⁎^
ECIe	0.93^⁎⁎^	0.73^⁎⁎^	0.91^⁎⁎^	0.77^⁎⁎^	0.76^⁎⁎^
CCCIp	0.70^⁎⁎^	0.70^⁎⁎^	0.74^⁎⁎^	0.68^⁎⁎^	0.67^⁎⁎^
ECIp	0.94^⁎⁎^	0.75^⁎⁎^	0.90^⁎⁎^	0.77^⁎⁎^	0.76^⁎⁎^

BST = body surface temperature; RT = rectal temperature; RR = respiration rate; HR = heart rate; PR = pulse rate; THI = Temperature-humidity index; IHTI = Iberia heat tolerance index; BHTI = Benezra heat tolerance index; THIn = thermal heat index; CCCIg = general comfort climatic conditions index; ECIg = general environmental comfort index; CCCIe = effective comfort climatic conditions index; ECIe = effective environmental comfort index; CCCIp = practical comfort climatic conditions index; ECIp = practical environmental comfort index; ** = significant (*p*<0.01).

## Discussion

### Climatological variables

The climatological data confirmed that the study captured the intended range of environmental variation, providing the necessary context to successfully characterize the adaptive profiles of Hararghe Highland (HH) sheep. Dry-season and afternoon air temperatures frequently exceeded the 25°C comfort threshold, particularly in lowland areas, while wet-season and morning conditions generally remained within the thermoneutral zone. This environmental variation allowed for a robust evaluation of whether HH sheep can maintain homeothermy under varying thermal loads. The observed significant changes in physiological parameters across different AT and RH values demonstrate the breed's ability to activate adaptive mechanisms in response to environmental challenges. Elevated AT combined with lower RH has been shown to alter physiological responses and may adversely affect animal productivity by influencing heat exchange with the surrounding environment (Furtado et al., 2017). Sheep are generally considered thermally comfortable when AT remains below 25°C and RH below 65% (Filho et al., 2011). In the present environment, temperature – not humidity was the primary limiting factor, as dry‑season and afternoon values frequently exceeded this threshold, particularly in lowland areas. The study revealed that different AT and RH values observed between seasons, periods, and agro-ecologies considered result in significant changes in physiological parameters measured in the HH sheep. The RH data showed higher values in the morning due to the lower AT values in the morning period of day; conversely, afternoon RH was lower as AT increased. The AT was higher in the dry season while RH was greater in the wet season. This result is in agreement with the report of Souza et al. (2014), who reported that in the dry season with higher AT, RH decreased, in contrast to the rainy season.

### Physiological parameter

Rectal temperature is widely recognized as the most reliable indicator of thermal stress, as it increases only when peripheral thermoregulatory mechanisms are insufficient to maintain homeothermy (Rashamol et al., 2018). In the present study, RT varied significantly, with consistently higher readings in the afternoon and during the dry season. This diurnal increase aligns with previous reports (Piccione et al., 2002; Torres et al., 2017), which attribute this pattern to the cumulative heat load from both metabolism and the environment. The lower absolute RT values recorded in HH sheep compared to the 39.1°C baseline suggested by Piccione et al. (2002) may be explained by the superior adaptive capacity of indigenous breeds; as suggested by Costa et al. (2018) in Morada Nova ewes, these breeds likely prioritize evaporative heat loss via panting to minimize core temperature elevation, a strategy supported by the concurrent rise in respiration rate observed in this study. Respiration rate represents the primary physiological response to heat stress, facilitating evaporative cooling through panting (Haile et al., 2019). Afternoon RR values were notably higher than morning values, with increased rates observed during the dry season and in lowland areas, indicating that panting is a key thermoregulatory strategy. Mohapatra et al. (2019) reported higher afternoon RR, PR, and RT in fat-rumped sheep during hot seasons, confirming that RR is a key indicator of heat stress, with afternoon temperatures leading to higher evaporative cooling demands. The present findings are consistent with Rathwa et al. (2017), who reported that high heat load causes a significant rise in RR as a key adaptation mechanism in native sheep. The elevated RR during the dry season is consistent with findings from Rathwa et al. (2017), who noted higher RR in summer than in winter among native sheep. The average RR in this study (23.9–28.8 breaths/min) was well below the low-stress level of 40–60 breaths/min suggested by Silanikove et al. (2000) and fell within the normal physiological range of 17–30 breaths/min suggested by Roger (2008).

In the present study, the average PR was 77.3 beats/min, with afternoon readings (80.3 beats/min) exceeding morning values (74.2 beats/min) (Table 3). This trend aligns with observations in Magra sheep, where PR rose from 71 beats/min under neutral conditions to nearly 100 beats/min during heat stress (Singh et al., 2016). The increase in PR during the dry season corroborates the findings of Rathwa et al. (2017), who reported higher PR in the summer than in the winter. The highest PR values observed in lowland areas (79.6 beats/min) indicate cardiovascular adaptations to thermal stress, similar to the findings of Torres et al. (2017), who reported elevated afternoon PR due to rising AT. The PR range of 74.2–80.3 beats/min (Table 3) is comparable to the 78.6–81.8 beats/min range reported by Rathwa et al. (2017) for indigenous sheep. Heart rate showed patterns that were similar to those of PR, with higher values in the afternoon, during the dry season, and in lowland areas. These cardiovascular changes, such as increased heart rate and blood flow, act as a primary mechanism to cope with heat stress, ensuring that heat is transported from the core to the surface, despite potential negative effects on animal productivity (Jumnake et al., 2024). Also elevated HR supports increased cardiac output, facilitating convective and radiative heat loss (Marai et al., 2007). The HR values recorded in this study (77.3–83.4 beats/min) were lower than those reported for Merino sheep under heat stress (90–108 beats/min; Wojtas et al. (2014) and for Dorper sheep (93.1 beats/min; Torres et al. (2017). These differences suggest that HH sheep maintain cardiovascular stability under heat stress compared to subtropical breeds. Body surface temperature was strongly influenced by diurnal and agro-ecological variation, with afternoon, dry season and lowland measurements significantly higher than morning, wet season and highland values. Body surface temperature (BST), were highly sensitive to environmental conditions, with afternoon, dry season and lowland measurements significantly higher than morning, wet season and highland values. Elevated BST reflects active peripheral vasodilation (Romanovsky, 2014), facilitating heat dissipation. Comparable seasonal increases in BST have been reported in fat rumped sheep (Mohapatra et al., 2019).

Compared with other indigenous and exotic sheep breeds, HH sheep maintained lower absolute rectal temperatures (37.4–38.5°C) than Merino sheep under heat stress (39.5–40.2°C) and lower heart rates than Dorper sheep, indicating reduced physiological strain under comparable thermal conditions. This demonstrates that HH sheep exhibit superior thermotolerance compared to commonly studied breeds, maintaining thermal stability with lower cardiovascular demand. Such a strategy is characteristic of well-adapted indigenous breeds and is clearly reflected in HH sheep, which maintained relatively stable rectal temperatures alongside moderate increases in respiratory and cardiovascular activity, indicating a reliance on efficient heat dissipation rather than elevation of core body temperature. This response pattern distinguishes HH sheep from less-adapted breeds that exhibit higher core temperature and greater physiological strain under similar thermal conditions.

### Heat stress indices

The simultaneous evaluation of climate-based and physiology-based indices provided additional insight beyond traditional single-index approaches. While climate-based indices such as THI and THIn effectively described the environmental thermal load, they did not fully capture the magnitude of physiological response in HH sheep. In contrast, physiology-based indices (ECIg, ECIe, and ECIp) showed stronger associations with key thermoregulatory variables such as BST and RR, indicating their greater sensitivity in reflecting the animal’s actual heat stress status. This integrated approach therefore allows a more comprehensive understanding of both external thermal conditions and internal adaptive responses, which would not be possible when relying on a single index alone. The temperature‑humidity index (THI) is a composite measure of AT and RH that enables objective evaluation of environmental conditions and is widely used in thermal stress research. In the present study, THI values increased significantly in the afternoon compared to the morning in both seasons. As shown in Table 4, THI values ranged from 20.9 ± 0.68 (wet‑season morning) to 26.8 ± 0.65 (dry‑season afternoon). According to the thresholds proposed by Marai et al. (2007), values below 22.2 °C indicate an absence of heat stress, while values between 22.2 and 23.3 °C signal the onset of heat stress. These findings indicate a shift from a thermoneutral state in the mornings to a state of heat stress by the afternoon. The THIn indices also showed a clear daily pattern, going from 63.9 ± 0.64 (wet-season morning) and 66.3 ± 0.73 (dry-season morning) to 68.6 ± 0.70 and 70.6 ± 0.73 in the dry-season afternoon, respectively (Table 4). Following the THIn classification (≤ 70) established by Mader et al., (2006) and Haile et al. (2019), morning values in both seasons and afternoon values during the wet season remained within the thermally comfortable range. On the other hand, the THIn in the afternoon during the dry season (70.6) was slightly above the comfort threshold, which means that the temperature was starting to become uncomfortable. Significant diurnal variation was observed for the IHTI across both seasons (*p*< 0.05), with the highest values recorded during the morning of the wet season (133.1; Table 3). These high IHTI values in the morning indicate that the animals were maintaining an optimal thermal comfort state, reflecting a high degree of physiological adaptation to the prevailing ambient conditions. According to Arero and Ozmen (2025), indigenous sheep breeds possess strong adaptive profiles, including morphological and physiological mechanisms that allow them to maintain homeostasis more effectively when environmental loads are low. The higher morning IHTI scores in this study further confirm that the sheep remained well within their thermoneutral zone, utilizing their native adaptive capacity to mitigate environmental stressors before the peak thermal loads of the afternoon. The BHTI values in the morning (2.98–3.14) were much higher than those in the afternoon (2.76–2.81; *p*< 0.05; Table 4). Although Benezra (1954) defines a score of 2.0 as the ideal adaptability threshold, the observed decline toward the afternoon suggests that the sheep effectively stabilized their physiological equilibrium as the day progressed. This resilience aligns with the high adaptive capacity and safety‑regulator mechanisms characteristic of indigenous sheep breeds in fluctuating environments Arero and Ozmen (2025). The heat stress indices CCCIg, CCCIe, and CCCIp exhibited significant variation (*p*< 0.05) across both time of day and season, with the highest values recorded during the afternoon of the dry season (28.0, 32.2, and 31.6, respectively). According to the classification criteria proposed by Silva et al. (2015 and Pantoja et al. (2018), environmental conditions during the morning period of both seasons fell within the range considered thermally comfortable for the animals. In contrast, the afternoon period of the dry season approached the threshold indicative of a potential risk zone. A similar diurnal pattern was observed for the ECIg, ECIe, and ECIp indices, which peaked at 37.6, 37.6, and 38.5, respectively, during the afternoon and showed statistically significant differences (*p*<0.05) compared to morning values (Table 4). Nevertheless, the interpretation of these indices suggests that the sheep remained within acceptable thermal comfort limits even during the afternoon period.

### Correlation between physiological variables and heat stress indices

The strong positive correlations (r = 0.63–0.94) between most heat stress indices (THI, THIn, CCCIg, CCCIe, CCCIp, ECIg, ECIe, ECIp) and physiological variables (BST, RT, RR, HR, PR) confirm that these indices reliably reflect the thermal load experienced by HH sheep and its associated physiological consequences. In particular, the environmental comfort indices (ECIg, ECIe, and ECIp) showed the highest associations with BST (r = 0.93–0.94) and RR (r = 0.90–0.91) (Table 5). These indices are mathematically derived from the physiological parameters they were correlated against, the high correlations likely reflect this mathematical dependency in addition to biological sensitivity. Nevertheless, this finding aligns with reports in buffaloes, where BST was identified as the most precise physiological variable for indicating thermal stress, and composite comfort indices demonstrated strong sensitivity to environmental changes (Pantoja et al., 2018). In contrast, the IHTI demonstrated exceptional negative correlations with RT (r = −1.00, p<0.01) and moderate to strong negative correlations with BST (r = −0.74), RR (r = −0.63), HR (r = −0.60), and PR (r = −0.61). These results are consistent with Pantoja et al. (2018), who reported that in male buffaloes, IHTI was the only index strongly and negatively correlated with RT (r = −1.00). Similarly, the BHTI showed significant negative correlations with all physiological variables, particularly with RR (r = −0.97), HR (r = −0.67), and PR (r = −0.66). Although all indices indicated increasing heat stress under elevated environmental temperatures, clear differences were observed in their performance. Climate-based indices such as THI and THIn were effective in describing environmental heat load but showed weaker associations with physiological variables. In contrast, ECI-based indices demonstrated stronger and more consistent relationships with BST and RR, indicating higher sensitivity in capturing actual physiological stress. This suggests that physiology-based indices provide a more accurate assessment of heat stress in HH sheep under field conditions. The significant correlations between physiological variables and the calculated heat stress indices (except IHTI and BHTI) support the applicability of these indices for quantifying the environmental thermal load experienced by HH sheep and its relationship with their physiological responses. While these indices serve as indicators of the thermal effect of the environment on animals, they do not directly measure animal comfort; rather, the thermal comfort status of HH sheep was inferred by interpreting index values alongside the corresponding physiological responses. According to Silva et al. (2015) and Pantoja et al. (2018), the correlations between physiological variables and heat stress indices indicate the sensitivity of each index in reflecting the animal's thermoregulatory responses to the environment. The correlation results, together with the classification thresholds of each index, indicate that THI, THIn, CCCIg, CCCIe, CCCIp, ECIg, ECIe, and ECIp are suitable for monitoring heat load and inferring the thermal status of HH sheep under the conditions of the present study. Although the ECI indices demonstrated superior predictive power, their calculation requires physiological measurement of BST, RT, and RR, which are less practical for routine field applications due to the need for animal restraint and specialized equipment. For on-farm use in Ethiopia, indices that require only AT and RH such as THI, THIn, and CCCIp are more feasible. These indices can be calculated using an inexpensive digital thermo-hygrometer, enabling livestock keepers to monitor heat stress risk in real time and implement preventive measures such as providing shade and adjusting grazing hours. This practical approach supports the sustainable management and conservation of locally adapted genetic resources under changing climate conditions. However, the limitations of this study, including short-term monitoring and small sample size, which may limit the generalizability of the findings. Therefore, future research should focus on long-term studies that integrate behavioral observations, genetic analyses, and improved management interventions to enhance understanding of the adaptive mechanisms of these sheep. Overall, the findings indicate that while traditional indices such as THI remain useful for environmental monitoring, physiology-based indices particularly ECIg and ECIe are more effective in evaluating animal-level heat stress and should be preferred when assessing thermoregulatory responses in HH sheep.

## Conclusions

This study demonstrates that HH sheep possess adequate thermoregulatory capacity to maintain homeothermy under the thermal conditions prevailing across three agro-ecological zones in eastern Ethiopia. The inclusion of the wet season provides insights into the full range of environmental challenges faced by these animals, emphasizing the importance of seasonality in identifying adaptive responses. In the dry season, HH sheep demonstrated a remarkable ability to maintain stable rectal temperatures despite rising ambient conditions, with moderate increases in respiratory rate (RR), heart rate (HR), and pulse rate (PR) reflecting the activation of physiological cooling mechanisms. These changes suggest that the breed effectively dissipates heat stress through respiratory and cardiovascular responses, thereby sustaining thermal balance without compromising homeothermy. The calculated heat stress indices, including THI, THIn, CCCIg, ECIg, CCCIe, CCCIp, ECIe, and ECIp, proved suitable for quantifying the environmental thermal load experienced by HH sheep and its associated physiological effects. Although THI is a useful indicator of environmental heat load, it does not directly measure animal comfort, which depends on physiological and behavioral responses. Future research should focus on genetic markers of thermotolerance, behavioral adaptations, and the development of user-friendly field devices to support wider application, particularly in resource-limited settings.

## Data availability

Data reported in this study are available upon request to the authors.

## Ethics statement

Ethical approval for this study was obtained from the School of Animal and Range Sciences Review and Approval Committee, Haramaya University, Ethiopia (Approval ID: SARS 051/24). The study was conducted as an observational field study and did not involve any invasive procedures on animals. Verbal informed consent was obtained from all participating livestock owners prior to the inclusion of their animals in the study.

## CRediT authorship contribution statement

**Mohammed Yousuf:** Writing – original draft, Methodology, Conceptualization. **Kefelegn Kebede:** Writing – review & editing, Supervision, Methodology. **Yesihak Yusuf:** Writing – review & editing, Supervision. **Gemeda Duguma:** Writing – review & editing. **Solomon Abegaz:** Writing – review & editing.

## Declaration of competing interest

The authors declare that they have no known competing financial interests or personal relationships that could have appeared to influence the work reported in this paper.
